# Invited Discussion on: Mechanical Thromboprophylaxis Alone in Body Contouring Surgery for Post Massive Weight Loss Patients: Is This Good Enough?

**DOI:** 10.1007/s00266-021-02459-1

**Published:** 2021-07-20

**Authors:** Eric Swanson

**Affiliations:** Swanson Center, 11413 Ash St, Leawood, KS 66211 USA

*Level of Evidence V* This journal requires that authors assign a level of evidence to each article. For a full description of these Evidence-Based Medicine Ratings, please refer to Table of Contents or online Instructions to Authors www.springer.com/00266.

Petersen et al. [1] report their experience using sequential compression devices without chemoprophylaxis in 64 body contouring surgery patients after massive weight loss. The authors report one (less than 1%) deep venous thrombosis (DVT). They conclude that mechanical prophylaxis using either compression stockings alone (41.5%) or in combination with sequential compression devices (58.5%) is effective in reducing the venous thromboembolism (VTE) risk. Petersen et al. [1] recognize that their study is not adequately powered to identify factors contributing to VTE risk. There is also no control group. Clinical diagnosis of DVT is notoriously unreliable [2]. Therefore, the true incidence of DVTs is unknown in this patient group.

Sequential compression devices hold an intuitive appeal. However, a 2012 Task Force of the American Society of Plastic Surgeons found insufficient evidence on which to make a recommendation [3]. Similarly, there is no evidence supporting the efficacy of compression stockings in plastic surgery patients [4].

To make informed decisions regarding treatment recommendations, one must first set aside preconceptions handed down from other specialties using antiquated diagnostic methods in patients undergoing joint replacements and abdominal surgery [2]. Second, a reliable diagnostic device (ultrasound) is needed. To learn more about VTEs developing after plastic surgery, Swanson initiated a 5-year prospective clinical trial of 1000 plastic surgery outpatients monitored with ultrasound surveillance [5]. Sequential compression devices were applied during the first half of the study, but not during the second half. The results were surprising: DVTs were rarely detected on scans the day after surgery. More often, DVTs were detected 1 week after surgery and were usually located, favorably, in the deep calf veins. With treatment (apixaban or rivaroxaban), these DVTs resolved in 5 weeks, on average. Notably, the use of sequential compression devices during surgery made no difference in VTE risk (0.9%). Moreover, a separate randomized laboratory study found no evidence of a fibrinolytic benefit from SCDs [6].

The 2011 VTE Prevention Study showed that VTE risk is spread over a range of Caprini scores in plastic surgery inpatients [7]. Almost as many VTEs (48%) occur in patients with scores less than 7 as in patients with scores of 7 or higher. This finding should not be surprising because Caprini scores are not based on relative risk [8]. Patients with scores of 7 or higher did not show a significant benefit from anticoagulation [7]. Despite equal (1.2%) incidences of VTEs in patients treated with or without enoxaparin, a marginal overall benefit (*p* = 0.042) for chemoprophylaxis was determined. This significant finding was produced by adjusting the data analysis to force the *p* value under 0.05 [5, 9]. The result, supporting chemoprophylaxis, has not been reproduced.

Counterintuitively, subsequent studies have even shown a reverse relationship—more VTEs occurring in anticoagulated patients [10, 11]. Regardless, a short course of anticoagulation after surgery is likely to be ineffective because the course is too early and too short to be effective in patients who are destined to develop DVTs, and potentially harmful for patients who are not [5].

Swanson’s study confirmed that several traditional VTE risk factors such as older age, number of procedures, operating time, and abdominoplasty, are significantly associated with an increased VTE risk [5]. However—and this finding was unexpected—this association disappears once the data analysis was corrected for age using logistic regression. Indeed, older patients are more likely to undergo abdominoplasties and combined procedures, which take longer to perform. There is a physical basis for this association; older patients have stiffer venous valves, causing local hypoxia and triggering DVTs [12]. Body mass index, hormonal supplementation, and smoking history do not significantly affect the VTE risk in plastic surgery outpatients [5].

Petersen et al. [1] completed all their cases in less than 3 hours [1]. Their only combination procedure was breast surgery and brachioplasty, although breast procedures are not listed among the types of procedures performed. Liposuction is not listed either. To expedite surgery, two surgeons and two assistants operated simultaneously. Is operating time an independent risk factor for VTEs? Hardy et al. [13] concluded that surgery that exceeds 3 hours poses additional risk. However, this association could not be isolated from confounding variables related to procedure complexity [14]. When these other variables are eliminated, such as in single-procedure series (facelifts, for example), this time correlation disappears [14]. Swanson generally limits operating times to less than 6 hours, not because this length of time holds special meaning but because it is a loose marker for the extent of surgery [14]. Patients quite understandably prefer to have one operation rather than two. Abdominoplasty, liposuction, and augmentation/mastopexy may be safely performed together by experienced surgeons [15]. Effective regional anesthesia and vasoconstriction using (warmed) wetting solutions help to limit blood loss and reduce systemic anesthesia and fluid requirements [15].

Anesthesia is an important, and often overlooked (it is not considered in the Caprini score), [2, 9] consideration. Petersen et al. [1] use general endotracheal anesthesia exclusively. By contrast, Swanson routinely performs surgery, including lower body lifts, on patients treated with intravenous propofol, a laryngeal mask airway, and no muscle relaxation (“SAFE” Anesthesia—Spontaneous breathing, Avoid gas, Face up, Extremities mobile) [9]. Total intravenous anesthesia reduces VTE risk [16]. Paralysis is avoided to preserve a functioning calf muscle pump during surgery [5, 16]. Prone positioning, with its additional anesthetic considerations, unfavorable body positioning, and unsuitability for combined breast surgery (usually done first to optimize sterility), is eliminated [9]. The objective is to disturb the patient’s physiology as little as possible during surgery [16]. A 6-hour total intravenous anesthetic in a spontaneously breathing patient is likely to be more physiologic than a 3-hour anesthetic in a patient who is intubated, paralyzed, prone, and receiving positive pressure ventilation and anesthetic gas. Postoperative side effects such as nausea and sedation are minimized [15]. In keeping with the emphasis on enhanced recovery, patients are discharged home after a mean recovery room stay of 51 minutes [15].

A multicenter randomized, controlled trial seems ideal [1]. However, such a study is not feasible for many reasons, including confounders, resistance of patients and providers to randomization, and the low incidence of this complication. Moreover, such a study would be unlikely to meet the ethical requirement of equipoise [17].

The authors are to be commended for thinking independently and not basing their VTE prophylaxis on conventional wisdom. Ultrasound surveillance allows the diagnosis to be made before the treatment, rather than the reverse [2, 5]. DVTs may be detected in evolution, and treated before they become dangerous [5]. This method avoids distressing bleeding problems created by anticoagulation [9]. This technology truly represents a win-win for patients and surgeons.
